# PAX1 represses canonical Wnt signaling pathway and plays dual roles during endoderm differentiation

**DOI:** 10.1186/s12964-024-01629-3

**Published:** 2024-04-26

**Authors:** Danxiu Miao, Jie Ren, Yanhan Jia, Yihui Jia, Yanshu Li, Huizhe Huang, Rui Gao

**Affiliations:** 1 Institute of Cardiovascular Diseases, Xiamen Cardiovascular Hospital, School of medicine, Xiamen University, Xiamen, 361000 China; 2 Department of Toxicology, College of Public Health, Harbin Medical University, Harbin, 150000 China; 3 Sichuan Cancer Hospital and Institute, Sichuan Cancer Center, School of Medicine, University of Electronic Science and Technology of China, Chengdu, 610000 China; 4 College of Public Health, Shantou University, Shantou, 515063 China; 5 The Second Affiliated Hospital of Chongqing Medical University, Chongqing, 400010 China

**Keywords:** PAX1, Wnt signaling pathway, TCF7L2, Endoderm differentiation, SCID

## Abstract

**Background:**

Paired box 1 (PAX1) is a transcription factor and essential for the development of pharyngeal pouches-derived tissues, including thymus. PAX1 mutations are identified in Severe Combined Immunodeficiency (SCID) patients with Otofaciocervical Syndrome Type 2 (OTFCS2). However, despite the critical roles of PAX1 in embryonic development and diseases, detailed insights into its molecular mode of action are critically missing.

**Methods:**

The repressing roles of PAX1 and SCID associated mutants on Wnt signaling pathway were investigated by luciferase reporter assays, qRT-PCR and in situ hybridization in HEK293FT, HCT116 cells and zebrafish embryos, respectively. Co-immunoprecipitation (co-IP) and western blotting assays were carried out to identify the molecular mechanisms underlying PAX1’s role on Wnt signaling pathway. hESC based endoderm differentiation, flow cytometry, high-throughput sequencing data analysis, and qRT-PCR assays were utilized to determine the roles of PAX1 during endoderm differentiation.

**Results:**

Here, we show that PAX1 represses canonical Wnt signaling pathway in vertebrate cells. Mechanically, PAX1 competes with SUMO E3 ligase PIASy to bind to TCF7L2, thus perturbing TCF7L2 SUMOylation level, further reducing its transcriptional activity and protein stability. Moreover, we reveal that PAX1 plays dual roles in hESC-derived definitive and foregut/pharyngeal endoderm cells, which give rise to the thymus epithelium, by inhibiting Wnt signaling. Importantly, our data show PAX1 mutations found in SCID patients significantly compromise the suppressing ability of PAX1 on Wnt signaling.

**Conclusions:**

Our study presents a novel molecular mode of action of PAX1 in regulation of canonical Wnt signaling and endoderm differentiation, thus providing insights for the molecular basis of PAX1 associated SCID, offering better understanding of the behavior of PAX1 in embryogenesis.

**Supplementary Information:**

The online version contains supplementary material available at 10.1186/s12964-024-01629-3.

## Background

Canonical Wnt signaling pathway is essential for embryogenesis and tissue homeostasis in multicellular organisms [1]. Perturbations in canonical Wnt signaling have been linked to a diverse set of human diseases, including congenital defects and various cancers [1]. The regulation of canonical Wnt signaling pathway is complicated and context-dependent in different tissues or cell types [2–4]. TCFs are the effectors of the Wnt cascade, which bind to their co-workers to activate or repress the transcription of target genes in a cell-type- and developmental stage-specific manner [5]. TCFs are a family of four members including TCF7, TCF7L1, TCF7L2 and LEF1 (also known as TCF1, TCF3, TCF4 and LEF1), displaying common structural features and are often expressed in overlapping patterns implying their redundancy [6]. TCF7L2 is the most intensely studied member and ubiquitously expressed in adult tissues, while other members are usually tissue-specifically expressed [6]. Thus, TCFs are spatiotemporally regulated and play their roles in a context-dependent manner. Moreover, tight regulation of TCFs by multiple post-translational modifications is one of the main switches that control canonical Wnt signaling [7]. Cumulative studies have indicated that SUMOylation affects the functions of protein substrates in various manners, including protein-protein interactions, subcellular localization, transcriptional activation and protein stability [8, 9]. SUMOylation of TCF7L2 has been shown to enhance its transcriptional activity and stimulate TCF7L2 mediated gene expression in canonical Wnt signaling pathway [10].

Definitive endoderm (DE) differentiation is an important biological process in early embryogenesis and gives rise to multiple organs including the respiratory and digestive tracts, thyroid, thymus, lungs, liver and pancreas [11]. In embryonic development, definitive endoderm forms during gastrulation from epiblast cells that ingresses through the primitive streak and undergoes an epithelial-to-mesenchymal transition (EMT) [12]. The developing gut tube, which derives from definitive endoderm, broadly segregates into four regions known as the anterior and posterior foreguts, midgut, and hindgut [13]. The anterior-most region of the foregut is known as the pharyngeal endoderm (PE), which forms the pharyngeal pouches [13]. Endoderm differentiation is dynamically orchestrated by a number of signaling pathways [14]. A growing number of evidence showed that canonical Wnt signaling pathway is critical for this process and suppression of Wnt signaling largely impairs definitive endoderm differentiation [14–17], whereas overactivated Wnt signaling in the later stage may disrupt endodermal pouch formation [13, 18].

PAX1 is a highly conserved transcription factor that belongs to the paired box-containing (PAX) gene family and plays crucial roles in diverse biological processes [19, 20]. PAX1 is mainly expressed in the skeleton system and pharyngeal pouches-derived tissues, such as thymus [20]. Importantly, recent studies identified quite some cases of PAX1 mutations associated Severe Combined Immunodeficiency (SCID) patients with Otofaciocervical Syndrome Type 2 (OTFCS2) [20–24]. Despite the critical roles of PAX1 in development and diseases, detailed insights into its molecular mode of action are critically missing.

In this study, we report for the first time that PAX1 acts as a negative regulator of canonical Wnt signaling pathway, mechanically by interacting with TCFs and decreasing the SUMOylation level of TCF7L2, thus reducing its transcriptional activity and protein stability. Ectopic expression of PAX1 leads to impaired differentiation of definitive endoderm in the early stage, while promotes foregut and pharyngeal endoderm formation in the later stage, negatively correlated with the roles of Wnt signaling during endoderm differentiation. Importantly, by using SCID associated PAX1 mutations as a departure point and investigating their functions in cellular model, we find that PAX1 variants found in SCID patients show compromised repression of TCF7L2; hence significantly weaken its function in regulation of Wnt signaling.

## Methods

### Cell culture and endoderm differentiation

HEK293FT cells and HCT116 human colon cancer cells were purchased from zqxzbio (ZQ0032) and Procell (CL-0096), respectively. They were maintained in DMEM containing L-glutamine (Gibco, 11,965,092) supplemented with 1 mM sodium pyruvate (Gibco, 11,360,070) and 10% fetal bovine serum (FBS) (Gibco, 10,270–106) in a humidified chamber at 37 °C with 5% CO_2_. Human embryonic stem cells H1 were obtained from WiCell (WA01) and were maintained on matrigel (Corning, 356,235) coated plates in mTeSR1 (STEMCELL technologies, 85,851) medium and passaged with Versene solution (Thermo Fisher Scientific, 15,040,066).

Endoderm differentiation was done according to previously published method [25]. Briefly, human H1 ESC lines were plated into 12-well plate to reach 80% confluency. Definitive endoderm differentiation was carried out in RPMI1640 medium (Gibco, 11,875,093) supplemented with increasing concentrations of KSR (Gibco, 10,828,028; 0% on day 1, 0.2% on day 2–3, and 2% on day 4–5). The following factors were added: Activin A, 100 ng/ml (R&D Systems, 338-AC; day 1–5); Wnt3a, 25 ng/ml (R&D Systems, 5036-WN; day 1). Anterior foregut endoderm (AFE) was induced in DMEM/F12 medium (Gibco, 21,331,020) supplemented with 0.5% B27 (Gibco, 17,504–044), 0.25uM all-trans retinoic acid (RA; Sigma, R2500), 50 ng/mL BMP4 (R&D Systems, 314-BP) and 5uM LY364947 (Selleck, S2805). Pharyngeal endoderm (PE) cells were differentiated in DMEM/F12 with 0.5% B27 plus 50 ng/mL Wnt3a, 0.1uM RA, 50 ng/mL BMP4, 5uM LY364947, 50 ng/mL FGF8b (R&D Systems, 423-F8) and 0.5uM KAAD-cyclopamine (MCE, HY-100535).

### Cell transfection, treatment and luciferase assay

HEK293FT or HCT116 cells were plated and grown until they reached around 70% confluency. Lipofectamine 2000 (Invitrogen, 11,668,019) was used for transfection following standard procedures. Total DNA amount used for transfection was kept the same using an empty pCMV5 vector throughout all the experiments.

10 μM CHIR99021 (Selleck, S2924) or 20 mM LiCl were added to the medium for 24 hours before being assayed to activate Wnt signaling. To inhibit proteasome complex, 10uM MG132 (MCE, HY-13259) was added to the cells for 6 hours before lysis. 100 μg/ml cycloheximide (CHX) (MCE HY-12320) was used to inhibit protein synthesis. Subasumstat (TAK981; MCE HY-111789) was added to the medium with indicated concentration for 48 hours to inhibit SUMOylation.

Luciferase assays were carried out according to the manufacturer’s recommendation using a dual luciferase kit (Promega, E1910). Ratios of Firefly luciferase versus Renilla luciferase were calculated, and minimum of three biological replicates were used to plot a graph with SDs. Individual experiments were repeated at least three times.

### Co-immunoprecipitation (co-IP) and western blotting

For co-immunoprecipitation, cells were lysed with lysis buffer (20 mM Tris pH 7.5, 1 mM EDTA, 1 mM EGTA, 150 mM NaCl, 1% Triton X-100) plus protease and phosphatase inhibitors on ice. After sonication and centrifugation, 10% of the supernatant was used for input (total cell lysate; TCL) and the left 90% for immunoprecipitation. Protein lysates were precleared with Protein G-Sepharose beads (GE Healthcare, 17–0618) at 4 °C on a rotary shaker for 1 h. Precleared lysates were incubated with M2 beads (Sigma, A2220) or prebinding dynabeads (Invitrogen, 10004D) with target antibody overnight at 4 °C. Protein complex bound beads were washed and resolved in a standard SDS-PAGE system.

Total cell lysates were quantitated by BCA assay (Thermo, 23,227). After boiling, equal amounts of protein extracts were separated by 12% SDS-PAGE and subsequently transferred to polyvinylidene difluoride (PVDF) membranes (Millipore). The membranes were blocked with 5% BSA for 1 h at room temperature (RT) and incubated with primary antibodies against flag (mouse anti-flag: Sigma F1804; Rb anti-flag: bioworld AP0007), HA (mouse anti-HA: Sigma H9658; Rb anti-HA: bioworld AP0005), MYC (Sigma, M4439), V5 (MBL, PM003), TCF7L2 (CST, 2569), ß-catenin (CST, 9562), TCF7 (CST, 2203), ß-actin (abcam, ab8227) and GAPDH (CST, 5174) in 5% BSA overnight at 4 °C. After washing for three times with TBST (0.242% Tris base, 0.8% NaCl, 0.1% Tween-20), the membranes were incubated with HRP-conjugated secondary antibodies (Jackson ImmunoResearch) for 1 h at room temperature. The target protein bands were detected using ECL solution (Advansta, K-12045-D50) and analyzed using ChemiDoc MP imaging system (BioRad).

For co-IP experiments in Fig. 3D and F, 4ug expression plasmid (1μg per construct) was transfected into HEK293FT cells in 6-cm dish per sample. Cells were harvested 48 hours later for co-IP experiments. Same amount of protein (around 2000μg) was used for immunoprecipitation and eluted in same volume of loading buffer after washing for each sample. 10% cell lysate was aliquoted for total cell lysate (input) and 10-20μg was loaded for western blotting. Densitometry quantification was performed with Image J.

### Immunofluorescence

Cells on coverslips were fixed with 4% Paraformaldehyde solution. Then Permeabilization was done with 0.25% Triton X-100 after 3 times washing with PBS. Cells were blocked for 1 h with 1% BSA in PBST (PBS+ 0.1% Tween 20) followed by incubation with anti-flag (Sigma, F1804) and HA (abcam, ab9110) antibodies overnight at 4 degree in a wet box. Cells were washed three times in PBS for 15 min and incubated with respective secondary antibodies (anti-mouse Alexa 488: Invitrogen, A11001; anti-rabbit Alexa 546: Invitrogen, A11035) along with DAPI for 1 h at room temperature. Antifade Mounting Medium (Beyotime, P0131) was used for mounting and image acquisition was done using an invitrogen EVOS FL Auto 2 fluorescent microscope.

### Flow cytometry analysis

Cells were dissociated into single cells with Accutase (Gibco, A1110501) and subsequently fixed with 1% paraformaldehyde for 20 minutes at room temperature (no fixation for cell surface marker) and stained with target antibodies (Alexa Fluor 647 Mouse anti-SSEA-4: BD Pharmingen 560,796; Alexa Fluor 488 Mouse anti-Human SOX17: BD Pharmingen 562,205) in PBS with 0.1% Triton X-100, 1% BSA and 10% horse serum after permeabilization and blocking. Data were collected on a LSR Fortessa cell analyzer (BD, 647794) and analyzed using BD FACSDiva Software. Fluorescence-activated cell sorting (FACS) gating was based on the corresponding isotype only antibody control.

### RNA isolation and quantitative real-time PCR (qRT-PCR) analysis

RNA was isolated using TRIzol (Invitrogen, 15,596,026) following the manufacturer’s instructions and cDNA synthesis was performed using High-capacity cDNA reverse transcription kit and random primers (Applied Biosystems, 4,368,813). The quantification of gene transcripts was measured by SYBR Select Master Mix (Applied Biosystems, 4,472,908) and StepOnePlus Real-Time PCR System (Applied Biosystems). *GAPDH* was used for normalizing gene expression in human cells and *rpl13a* was used for normalization in zebrafish embryos. Primers used for qRT-PCR are listed in Supplementary Table S1.

### RNA-seq and ChIP-seq data analysis

Total RNA was isolated with TRIzol (Invitrogen, 15,596,026) following the manufacturer’s instructions. RNA quality was checked with Fragment Analyzer 2100 (Agilent). Optimal Dual-mode mRNA Library Prep Kit was used for library preparation. Samples were sequenced on DNBSEQ-T7 sequencer (MGI). Basic data processing was performed with Galaxy. Briefly, the FASTQ files were mapped to Human Genome, GRCh38 with TopHat2. The outputted binary version of a Sequence Alignment/Map (BAM) files were used to get the expression count table produced by featureCounts. Annotation file (hg38.refGene.gtf.gz) was downloaded from UCSC.edu. Differential expression (log2 fold change ≤ − 1, ≥1; *p*-value < 0.05) was quantified and normalized using DESeq2. Heatmap2 tool was used for heatmap generation.

TCF7, TCF7L1, TCF7L2 and LEF1 ChIP-seq datasets (GSE182842) were obtained from NCBI GEO database. ChIP experiments were done in human iPS72.3 cells derived definitive endoderm cells. ChIP-seq reads were aligned to the hg19 genome using bowtie2. Bigwig (bw) files downloaded from the datasets were visualized and analyzed with Integrative Genomics Viewer (IGV).

### Zebrafish embryo analysis

Zebrafish (*Danio rerio*, AB strain) was kept at 28.5 °C under a light and dark cycle of 14 and 10 hours, respectively. Fish staging and embryo production were carried out as described in [26]. Microinjection of mRNAs and morpholino (MO) oligonucleotides, and in situ hybridizations were performed as previously described [27]. Capped mRNAs were synthesized using the mMESSAGE mMACHINE Kit (Ambion AM1344) according to the manufacturer’s instructions. The concentration of mRNA was measured by Multiskan SkyHigh Microplate Spectrophotometer (Thermo Fisher Scientific). Morpholinos were ordered from Gene Tools and prepared in stocks. The sequence of the *pax1a* MO is 5′-TGTTTGCTCCATTTGCTTTTGCGAT-3′ [28] and *pax1b* MO is 5′-CCCGTGTCTCCCGCTAAAGACTGCC-3′ [27]. 100 pg *pax1* capped mRNA (50 pg *pax1a* + 50 pg *pax1b*) or 8 ng *pax1* MO (4 ng *pax1a* MO + 4 ng *pax1b* MO) were injected in 4 nL per zebrafish embryo into the yolk at the one-cell stage. Embryos were raised to the indicated stages and harvested randomly for each experiment. All Institutional and National Guidelines for the care and use of animals were followed.

### Statistical analysis

All experiments were performed at least two or three independent times and respective data were used for statistical analysis. Data are presented as mean ± SD. Differences between groups were assessed using unpaired two-tailed Student’s t-test. Statistical significance was defined as follows: *P < 0.05, **P < 0.01, ***P < 0.001 or #*P* < 0.05, ##*P* < 0.01, ###*P* < 0.001 in Fig. 7B and Fig. 7C.

## Results

### PAX1 acts as a negative regulator of canonical Wnt signaling pathway

**Fig. 1:**
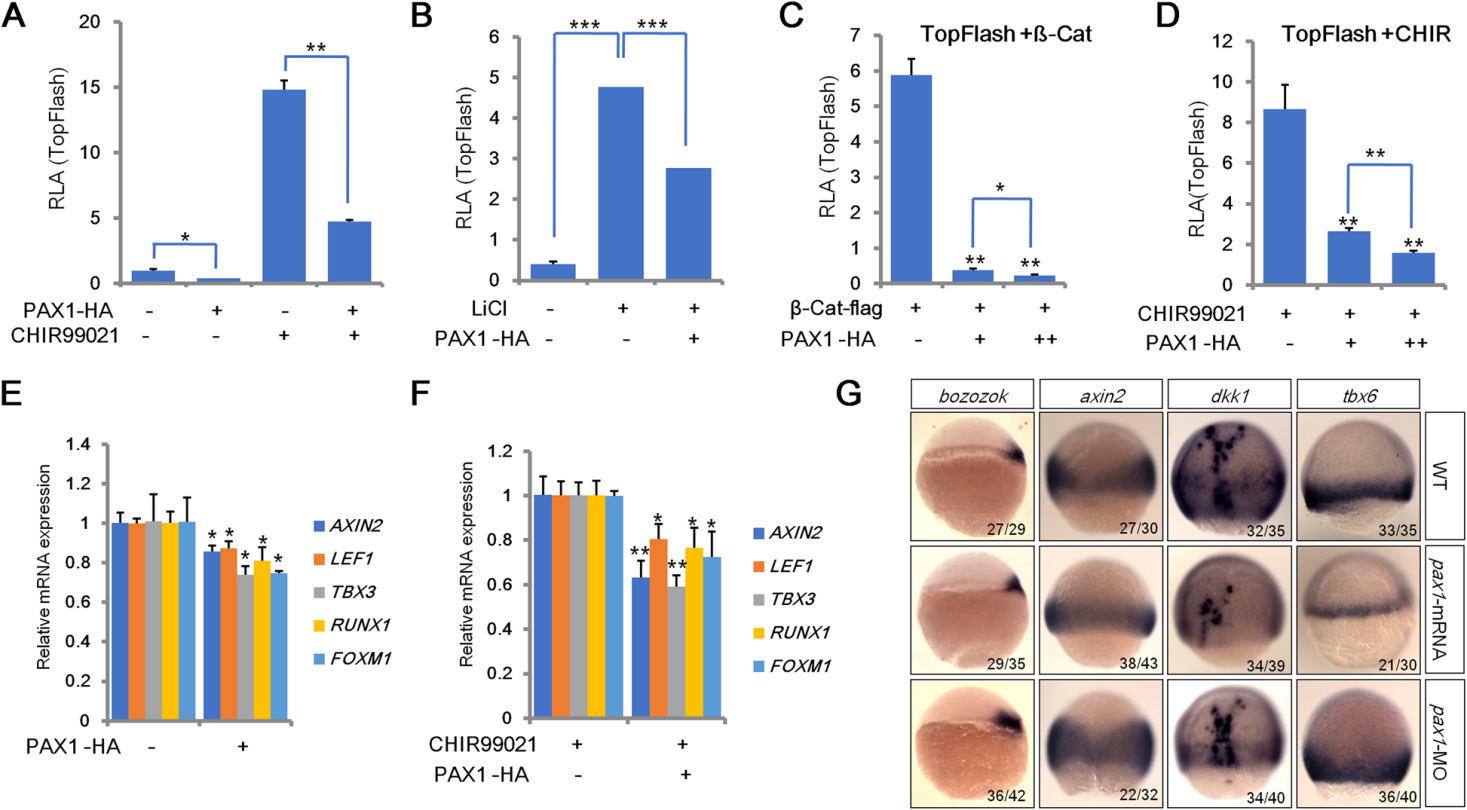
PAX1 plays a negative role in canonical Wnt signaling pathway. **A-B** TopFlash luciferase reporter assays in HEK293FT cells transfected with an empty vector control or a PAX1 expression plasmid, with or without treatment of GSK3 inhibitor CHIR99021 (CHIR) (A) or LiCl (B). RLA: relative luciferase activity. **C-D** TopFlash luciferase reporter assays in HEK293FT cells with transfection of an increasing dose of HA-tagged PAX1 and activated Wnt signaling by co-transfection of flag-tagged β-catenin (β-Cat) (C) or treatment with CHIR99021 (D). **E-F** Quantitative real-time PCR (qRT-PCR) analyses of Wnt target genes performed in HEK293FT cells with or without CHIR99021 treatment. **G** Expression of Wnt target genes by in situ hybridization in WT (wild type) control, *pax1* mRNA or morpholino (MO) injected zebrafish embryos. The frequency of embryos with the indicated patterns is shown in the bottom right corner of each group. Bar graphs and error bars represent mean ± standard deviation (SD) of at least three biologically independent experiments. *P < 0.05; **P < 0.01; ***P < 0.001. This is the same in the following figures unless specifically indicated

To investigate the roles of PAX1 in regulation of canonical Wnt signaling pathway, luciferase reporter assays were performed in HEK293FT cells with TopFlash reporter harboring TCF-binding sites. As shown in Fig. 1, PAX1 inhibits TopFlash activity significantly with or without treatment of GSK3 inhibitors CHIR99021 (Fig. 1A and S1A) or lithium chloride (LiCl) (Fig. 1B and S1B). Similar results were observed with an increasing dose of PAX1 when Wnt signaling is activated by ß-catenin or CHIR99021 (Fig. 1C, D and S1C, S1D). To confirm these results, we used qRT-PCR assays to check the expression of Wnt target genes in HEK293FT cells. Consistent with the results of TopFlash report assays, PAX1 overexpression (OE) in HEK293FT cells significantly decreases the expression levels of Wnt target genes, such as *AXIN2* [29, 30]*, LEF1* [31]*, TBX3* [32]*, RUNX1* [33] and *FOXM1* [34] when cells were treated with or without CHIR99021 (Fig. 1E-1F). To extend our findings beyond HEK293FT cells, TopFlash reporter and qRT-PCR assays were also performed in HCT116 cells to assess the potential effect of PAX1 on Wnt signaling (Fig. S1E-S1H). Consistently, our results showed significant repression of TopFlash activity by PAX1 in HCT116 cells as well (Fig. S1F-S1G). Downregulation of Wnt target genes including *EPHB2* [35]*, RUNX2* [36] and *JUN* [37], was also observed in HCT116 cells (Fig. S1H). To test whether pax1 plays a consistent role on Wnt signaling pathway in vivo, microinjection of *pax1* morpholino (MO) or *pax1* mRNA was utilized to knock down or overexpress *pax1* in zebrafish embryos [27, 28]. Results of in situ hybridization assays showed that pax1 represses the expression of wnt target genes such as *bozozok* [38]*, axin2* [29, 30]*, dkk1* [39] and *tbx6* [40] in vivo (Fig. 1G). Similarly, we also observed significant suppression of wnt target genes including *ccnd1* [41, 42]*, lef1* [31]*, sox17* [43] and *col12a1a* [44] by pax1 in zebrafish embryos analyzed by qRT-PCR (Fig. S1I). Taken together, these results indicated that PAX1 acts as a negative regulator in Wnt signaling pathway.

### PAX1 interacts with TCFs

**Fig. 2:**
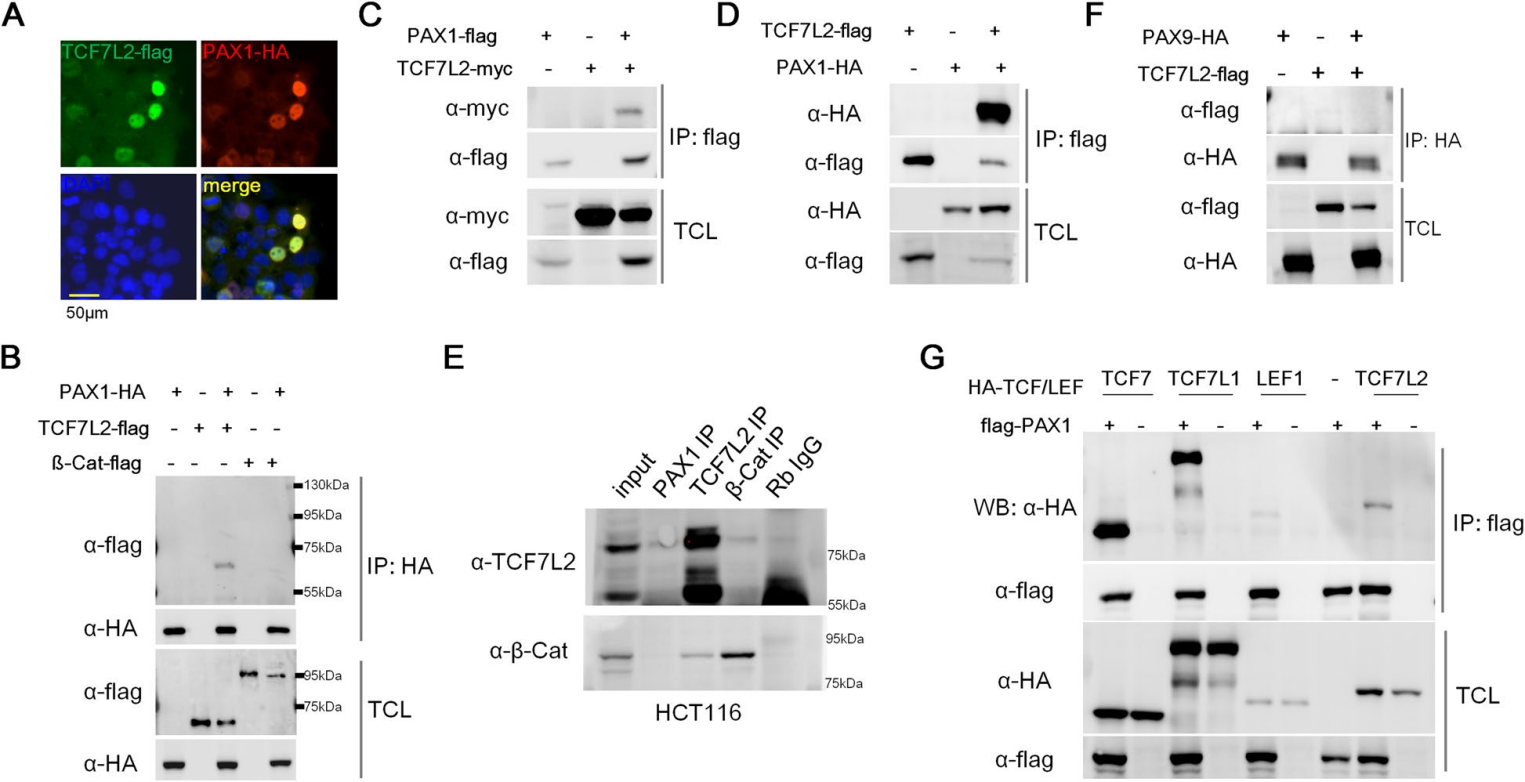
PAX1 interacts with TCF7L2. **A** Immunofluorescence showed the subcellular localization of TCF7L2 and PAX1 by co-transfection of TCF7L2-flag and PAX1-HA tag fused expression plasmids in HEK293FT cells. DAPI staining revealed the nuclei. Merged image showed that TCF7L2 and PAX1 are co-localized in the nucleus. **B-D** PAX1 and TCF7L2 or β-catenin (β-Cat) expression plasmids were transfected alone or together into HEK293FT cells and their interaction was determined by co-immunoprecipitation (co-IP) and western blotting. TCL: total cell lysate. **E** The endogenous interaction of PAX1 and TCF7L2 was tested in HCT116 cells. β-Cat and TCF7L2 IPs were used as positive controls. **F** PAX9 and TCF7L2 expression plasmids were transfected alone or together into HEK293FT cells and their interaction was determined by co-IP and western blotting. **G** The interactions between PAX1 and TCF/LEF family members were assessed by co-IP assays and western blotting, with PAX1 and TCFs expression plasmids transfection alone or together into HEK293FT cells

To address the molecular mechanism by which PAX1 inhibits canonical Wnt signaling pathway, firstly we tested the subcellular localization of PAX1. As a transcription factor, PAX1 co-localized with TCF7L2 in the nucleus as expected (Fig. 2A). Thus, we tested whether PAX1 interacts with ß-catenin or TCF7L2, which are two main effectors of canonical Wnt signaling pathway in the nucleus. Interestingly, we found PAX1 interacts with TCF7L2, but not with ß-catenin (Fig. 2B-2D). Same results were shown in HCT116 cells with endogenous co-immunoprecipitation (co-IP) (Fig. 2E). The endogenous interaction between TCF7L2 and ß-catenin was used as a positive control (Fig. 2E). As we can see from the result, PAX1 interacts with TCF7L2 endogenously in HCT116, comparable to ß-catenin (Fig. 2E). In addition, as a gene from the same subfamily with PAX1, PAX9 was reported to play a role in modulating Wnt signaling during murine palatogenesis [45]. So we want to test whether PAX9 also interacts with TCF7L2 as PAX1 does. Co-IP result showed that PAX9 does not interact with TCF7L2 (Fig. 2F), indicating that PAX1 plays its role in regulating canonical Wnt signaling differently with PAX9. To determine whether PAX1 interacts with other members of TCF/LEF family, flag-tagged PAX1 together with HA-tagged TCF7, TCF7L1, as well as LEF1 expression plasmids were transfected into HEK293FT cells and co-IP results showed interactions between PAX1 and all the TCF/LEF family members (Fig. 2G), suggesting PAX1 may regulate canonical Wnt signaling pathway via its interactions with TCFs.

To study this question in a more comprehensive manner, we generated a series of deletion constructs of TCF7L2 and PAX1 to identify which domains participate in their functional interaction (Fig. S2A and S2C). Co-IP in HEK293FT cells showed that TCF7L2 mutants lacking the β-catenin-binding domain (ΔβBD: amino acids 1–55 deleted), N-terminal domain (ΔN: amino acids 1–236 deleted) or middle domain harboring the Groucho/TLE-binding domain (ΔM: amino acids 236–326 deleted) are comparable with full-length TCF7L2 in binding with PAX1 (Fig. S2B). In contrast, TCF7L2 constructs lacking the high-mobility group (HMG) box DNA-binding domain (ΔHMG: amino acids 326–393 deleted) or its C-terminal domain (ΔC: amino acids 393–455 deleted) result in diminished interaction with PAX1 (Fig. S2B), demonstrating the importance of these two domains for the interaction between TCF7L2 and PAX1. Moreover, PAX1 deletion mutants without its N-terminal domain (ΔN: amino acids 1–98 deleted), the paired box domain (ΔP: amino acids 98–234 deleted), the middle domain (ΔM: amino acids 234–290 deleted), the octapeptide domain (ΔO: amino acids 290–297 deleted), or the C-terminal domain (ΔC: amino acids 297–458 deleted) alone do not affect the binding with TCF7L2 too much, while absence of the N-terminal domain, the paired box domain and the middle domain together (OC: amino acids 1–290 deleted) almost abolishes the interaction with TCF7L2 (Fig. S2D), indicating the half part at the N-terminal of PAX1 (amino-acid 1–290) is responsible for the binding of PAX1 with TCF7L2.

### PAX1 inhibits the interaction between TCF7L2 and PIASy, and thus reduces the SUMOylation of TCF7L2

**Fig. 3:**
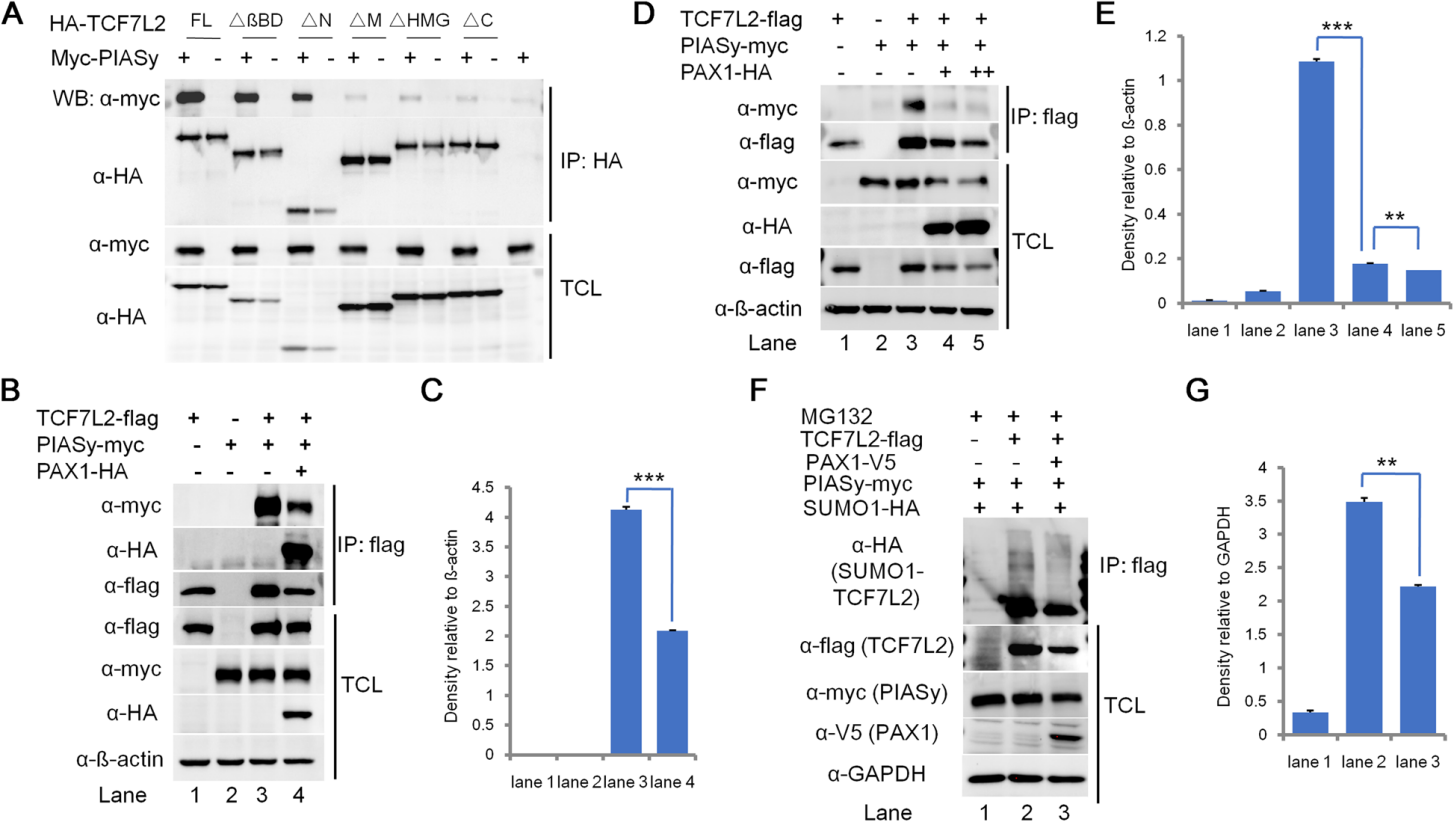
PAX1 inhibits the interaction between TCF7L2 and PIASy, and decreases the SUMOylation level of TCF7L2. **A** PIASy interacts with the middle, HMG and C-terminal domains of TCF7L2. Different HA-tagged TCF7L2 deletion constructs depicted in Fig. S2A were transiently transfected into HEK293FT cells together with Myc-tagged PIASy. Cell lysates were immunoprecipitated with M2 beads (anti-flag), followed by western blotting using anti-HA for TCF7L2 proteins and anti-Myc for PIASy. **B** Interactions between TCF7L2 and PIASy were assessed by co-IP and western blotting with empty vector control or PAX1-HA cotransfection. HEK293FT cells were transfected with flag-tagged TCF7L2 together with PIASy-myc and PAX1-HA plasmids. Following co-IP with M2 beads, TCL and IP samples were assayed by western blotting using anti-myc antibody for PIASy and anti-HA antibody for PAX1, respectively. ß-actin was used as loading control. **C** Densitometry analysis of western blots in (B) (*n* = 2, mean ± SD. *P < 0.05, **P < 0.01, ***P < 0.001). **D** PAX1 disturbs the interaction between TCF7L2 and PIASy in a dose-dependent manner. Same as (B) except increasing amount of HA-PAX1 (0 μg, 1 μg, and 2 μg) were transfected into HEK293FT cells together with flag-TCF7L2 and myc-PIASy. When flag-tagged TCF7L2 was immunoprecipitated by M2 beads (anti-flag), the amount of coprecipitated Myc-tagged PIASy was negatively correlated with the dose of cotransfected PAX1. **E** Densitometry analysis of western blots in (D) (n = 2, mean ± SD. *P < 0.05, **P < 0.01, ***P < 0.001). **F** Western blotting analysis of SUMOylation level of TCF7L2. HEK293FT cells were cotransfected with plasmids expressing flag-TCF7L2, V5-PAX1, and HA-tagged SUMO1. Cells were treated with proteasome inhibitors MG132 (10 μM) for 6 hours before harvested. GAPDH was used as loading control. **G** Densitometry analysis of western blots in (F) (n = 2, mean ± SD. *P < 0.05, **P < 0.01, ***P < 0.001)

To further investigate the molecular mechanisms underlying the regulation of PAX1 on Wnt signaling, we tested whether the interaction between PAX1 and TCF7L2 affects the binding of TCF7L2 with its other working partners. Considering that PIASy (also known as PIAS4), a SUMO E3 ligase essential for TCF7L2 SUMOylation [10], binds TCF7L2 at its middle domain, HMG box domain and C-terminal domain (Fig. 3A), covering the same domains of TCF7L2 binding with PAX1 (Fig. S2B), we wanted to test whether PAX1 competes with PIASy by binding the HMG box and C-terminal domains of TCF7L2. Co-IP assay showed that PAX1 overexpression disrupts the interaction between TCF7L2 and PIASy remarkably compared with control group (Fig. 3B and C), which is also in a dose-dependent manner (Fig. 3D and E). Thus, we hypothesized PAX1 may affect the SUMOylation of TCF7L2. To confirm our hypothesis, SUMOylation assay was done with empty vector (as control) or PAX1 expression plasmid. The results showed that PAX1 ectopic expression leads to significant decrease of TCF7L2 SUMOylation level (Fig. 3F and G). Taken together, these data suggested that one of the mechanisms whereby PAX1 inhibits Wnt signaling is reducing the SUMOylation of TCF7L2.

### PAX1 reduces TCF7L2 protein stability

**Fig. 4:**
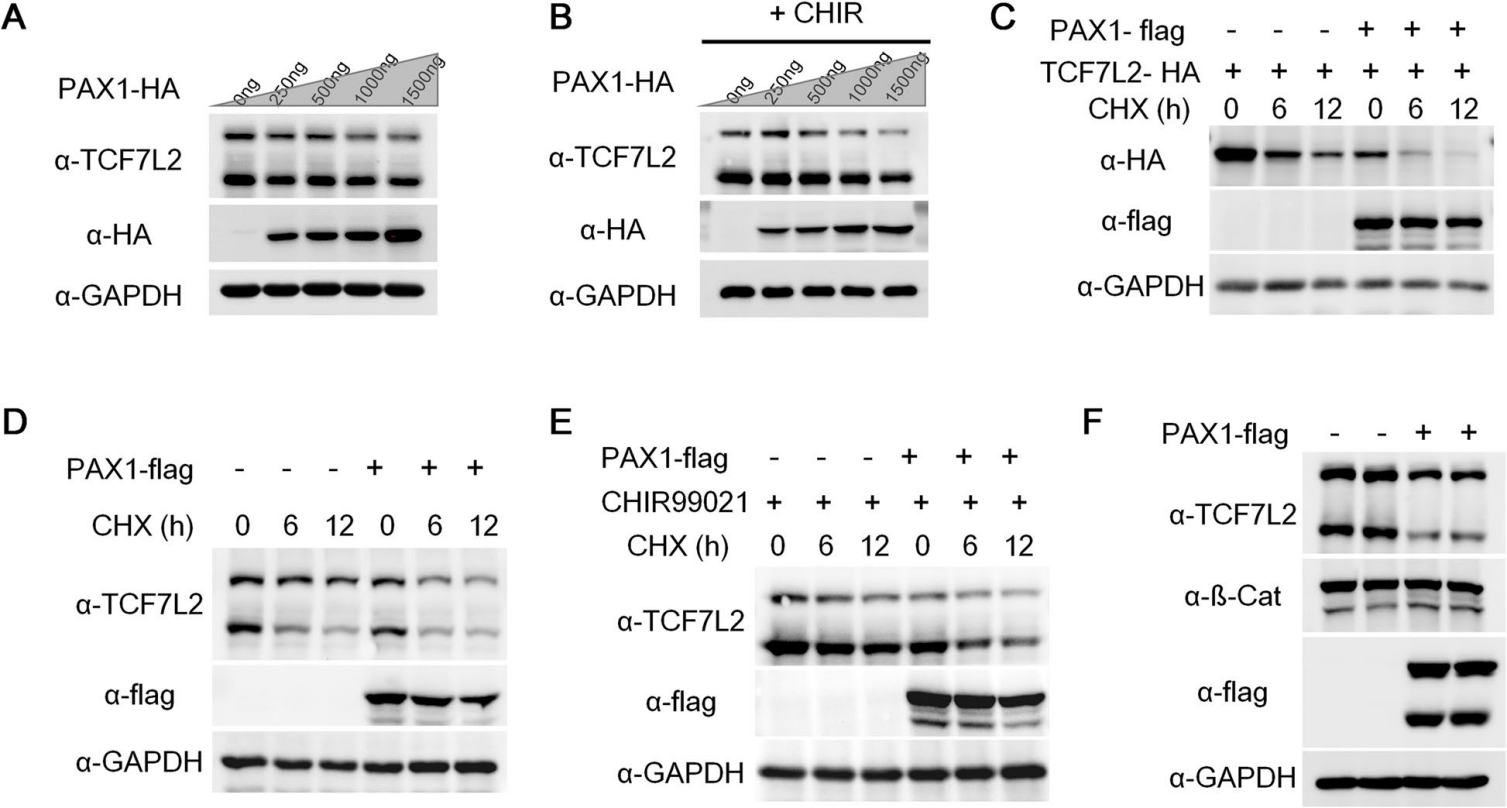
PAX1 reduces TCF7L2 protein stability. **A** HEK293FT cells were transfected with increasing amount of PAX1-HA (0, 250, 500, 1000, 1500 ng) expression plasmid and endogenous TCF7L2 levels were analyzed by western blotting. Both bands shown in TCF7L2 blot indicate different splicing isoforms of human TCF7L2 expressed in HEK293FT cells. GAPDH was used as loading control. **B** Same as (A) except cells were treated with 10 μM CHIR99021 (+CHIR) for 24 hours before harvested. **C** HEK293FT cells were transfected with TCF7L2-HA and empty vector (−) or PAX1-flag plasmid (+) and 2 days later were treated with 100 μg/ml cycloheximide (CHX) for indicated time and harvested. The expression of TCF7L2 (HA), PAX1 (flag), and GAPDH were analyzed by western blotting. **D** HEK293FT cells were transfected with empty vectors (−) or PAX1-flag plasmids (+) and cells were treated with 100 μg/ml CHX for indicated time before harvested. Endogenous TCF7L2 levels were analyzed by western blotting. GAPDH was used as loading control. **E** Same as (D) except cells were treated with 10 μM CHIR99021 (CHIR) for 24 hours before harvested. **F** Western blotting to analyze the protein levels of indicated proteins in WT and PAX1 overexpression (OE) HCT116 cell lines. GAPDH was used as loading control. Both bands detected in the flag blot should be PAX1 isoforms (NCBI database: NP_006183.2 and NP_001244025.1) which contains 534 and 457 amino acids respectively

SUMOylation of target proteins could change their transcriptional activity and protein stability [8, 9]. As we can see from the data of luciferase reporter assays with TopFlash, which contains the binding sites of TCF7L2, PAX1 reduces TopFlash signals robustly with different treatments (Fig. 1A-D), suggesting that PAX1 inhibits the transcriptional activity of TCF7L2. Next we wanted to examine whether PAX1 ectopic expression affects the protein stability of TCF7L2. Firstly we checked the protein levels of TCF7L2 with increasing dose of PAX1 and observed significant reduction of TCF7L2 in a dose-dependent manner with or without CHIR99021 treatment (Fig. 4A B and S4A-4B). To examine whether PAX1 reduces the protein stability of TCF7L2, empty vector (EV) or PAX1 expression plasmids were cotransfected with TCF7L2 expression construct into HEK293FT cells and the protein levels of TCF7L2 under the treatment of cycloheximide (CHX) at different time points were tested. The data showed the half-life of TCF7L2 protein is obviously shortened by PAX1 (Fig. 4C-E and S4C-4E). Furthermore, we tested TCF7L2 protein levels in HCT116 cells as well. A clear reduction of TCF7L2 level was observed while no changes of ß-catenin (Fig. 4F). Altogether, these data demonstrated that PAX1 decreases TCF7L2 protein level by reducing its stability.

In addition, we further examined the level of TCF7L2 by employing a SUMO inhibitor (TAK981) treatment (Fig. S4F-S4H). TCF7L2 protein level is indeed reduced when its SUMOylation is inhibited with 5uM or 10uM TAK981 treatment for 48 hours (Fig. S4F). Interestingly, we observed that TCF7L2 protein level still show obvious decrease as a result of PAX1 overexpression when treated with TAK981 (Fig. S4G-S4H), suggesting that PAX1 may regulate TCF7L2 not only through inhibiting its SUMOylation, but also through other mechanisms which need further investigation.

### PAX1 inhibits Wnt signaling pathway during endoderm differentiation

As is well known, PAX1 is a highly conserved transcription factor and essential for embryonic development. During mouse embryonic development, *Pax1* transcripts can be detected as early as E8.5 in the ventromedial part of newly formed somites when they are undergoing de-epithelialization, as well as in the endoderm of the foregut region examined by in situ hybridization [20, 46, 47]. To investigate whether PAX1 regulates canonical Wnt signaling pathway during embryogenesis, we firstly examined the expression of PAX1 in different cell populations in the published scRNA-seq data from the isolated anterior cardiac region of mouse embryos during E7.75 to E8.25 [48]. Thanks to new techniques, we can observe that *Pax1* is clearly expressing in the definitive endoderm in the early stage of mouse embryonic development (Fig. S3A), and later in foregut endoderm as shown in another scRNA-seq data obtained in fluorescence-activated cell sorting (FACS) purified mouse gut tube at the 9-somite stage (E8.5) [49].

**Fig. 5:**
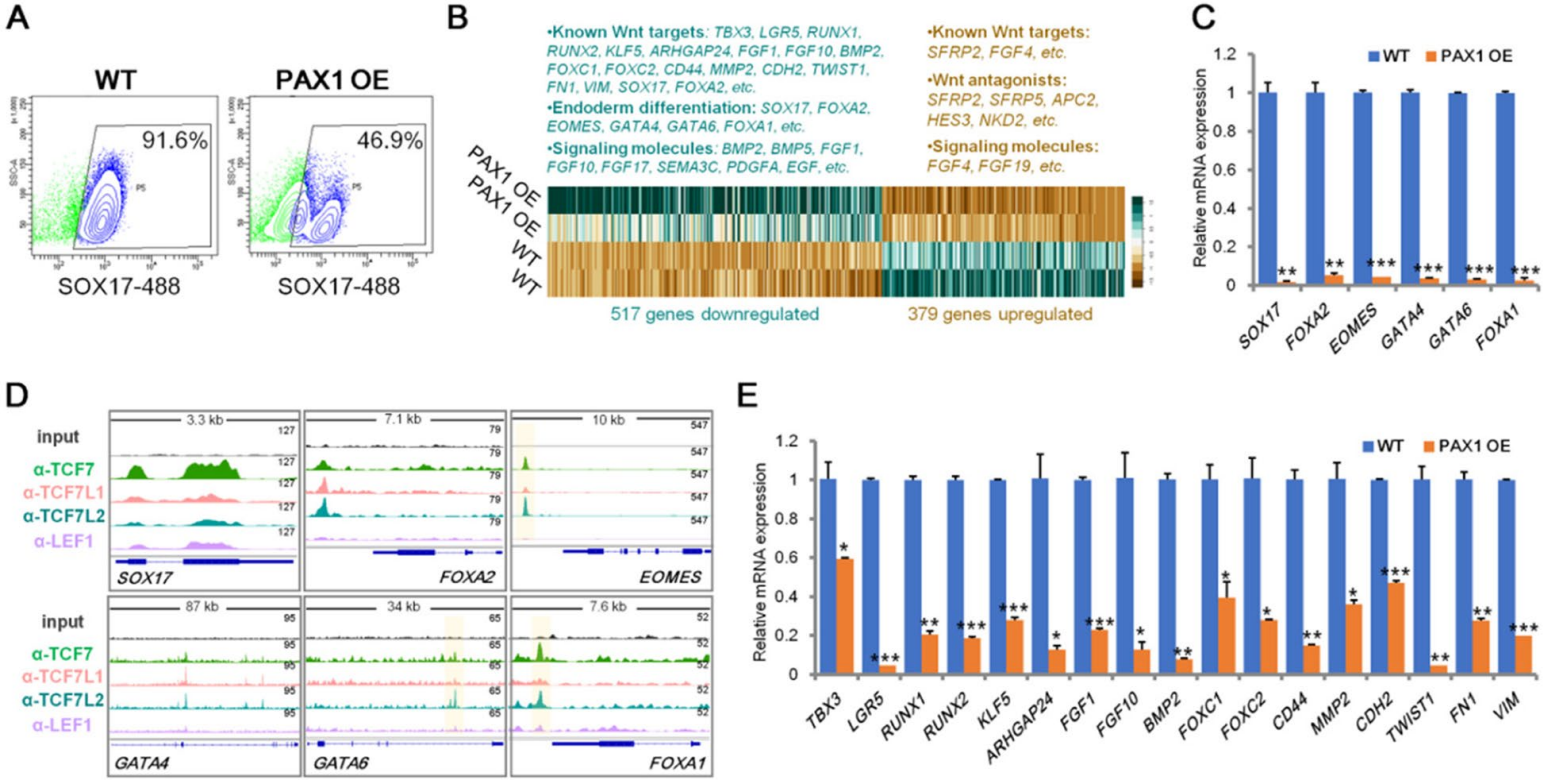
PAX1 ectopic expression leads to impaired definitive endoderm differentiation by inhibiting Wnt signaling. **A** Flow cytometry analysis of wild-type (WT) control and PAX1 overexpressed (OE) hESC-derived definitive endoderm cells stained for SOX17 (Alexa Fluor 488) demonstrates decreasing of SOX17^+^ population when PAX1 was ectopically expressed for a representative differentiation (*n* = 3 independent differentiations). **B** Heatmap representation of RNA-seq analysis of definitive endoderm cells derived from WT and PAX1 OE hESCs (n = 2; log2 fold change ≤ − 1, ≥1; *p*-value < 0.05). Differentially expressed known Wnt targets, endoderm markers and signaling molecules were listed above. **C** qRT-PCR analyses of endoderm marker genes in hESC-derived definitive endoderm cells. **D** Genome tracks of TCF7, TCF7L1, TCF7L2 and LEF1 ChIP-seq reads analyzed from datasets (GSE182842) on definitive endoderm marker genes in human pluripotent stem cell-derived definitive endoderm cells. **E** qRT-PCR analyses of known Wnt target genes in hESC-derived definitive endoderm cells

To study the roles of PAX1 in human endoderm differentiation, we generated a human embryonic stem cell (ESC) line with PAX1 overexpression (OE) (Fig. S3B). Firstly, we tested whether PAX1 OE affects the identity of ESCs. Cell cytometry analysis of human ESC surface marker SSEA4 and qRT-PCR analysis of the expression of pluripotency marker genes including *POU5F1*, *SOX2* and *NANOG* showed that PAX1 OE does not change the ESC pluripotency identity (Fig. S3C-S3D). Using an established protocol [25], we differentiated WT and PAX1 OE ESC lines to definitive endoderm stage (Fig. S3E) and observed that PAX1 OE significantly represses definitive endoderm differentiation as shown by cell cytometry analysis of definitive endoderm marker SOX17 (Fig. 5A). To reveal the molecular mechanism underlying the action of PAX1 in definitive endoderm differentiation, we did RNA-seq analysis and qRT-PCR confirmation in hESC-derived definitive endoderm cells (Fig. 5 and Supplementary Table S2). Consistent with the impaired definitive endoderm differentiation we observed (Fig. 5A), the expression levels of endoderm development associated genes such as *SOX17, FOXA2, EOMES, GATA4, GATA6* and *FOXA1,* bound by TCFs in definitive endoderm cells (Fig. 5D)*,* are dramatically decreased with PAX1 ectopic expression (Fig. 5B and C). In parallel, the levels of mesenchymal marker genes including *FOXC2, CD44, MMP2, CDH2, TWIST1, FN1* and *VIM* are also reduced (Fig. 5B and E), suggesting that the EMT, which is an essential process for definitive endoderm formation [12, 50, 51], is disturbed by PAX1. Importantly, we also found PAX1 OE leads to significant downregulation of a subset of known Wnt target genes, including *TBX3* [32]*, LGR5* [52]*, RUNX1* [33]*, RUNX2* [36]*, KLF5* [53]*, ARHGAP24* [54]*, FGF1* [55]*, FGF10* [56, 57]*, BMP2* [58]*, FOXC1* [59]*, FOXC2* [60]*,CD44* [61]*, MMP2* [62]*, CDH2* [63, 64]*, TWIST1* [65, 66], *FN1* [64, 67, 68] and *VIM* [69] (Fig. 5B and E). Altogether, these results support our finding that PAX1 plays a repressing role in Wnt signaling pathway and thus restrains definitive endoderm differentiation.

**Fig. 6:**
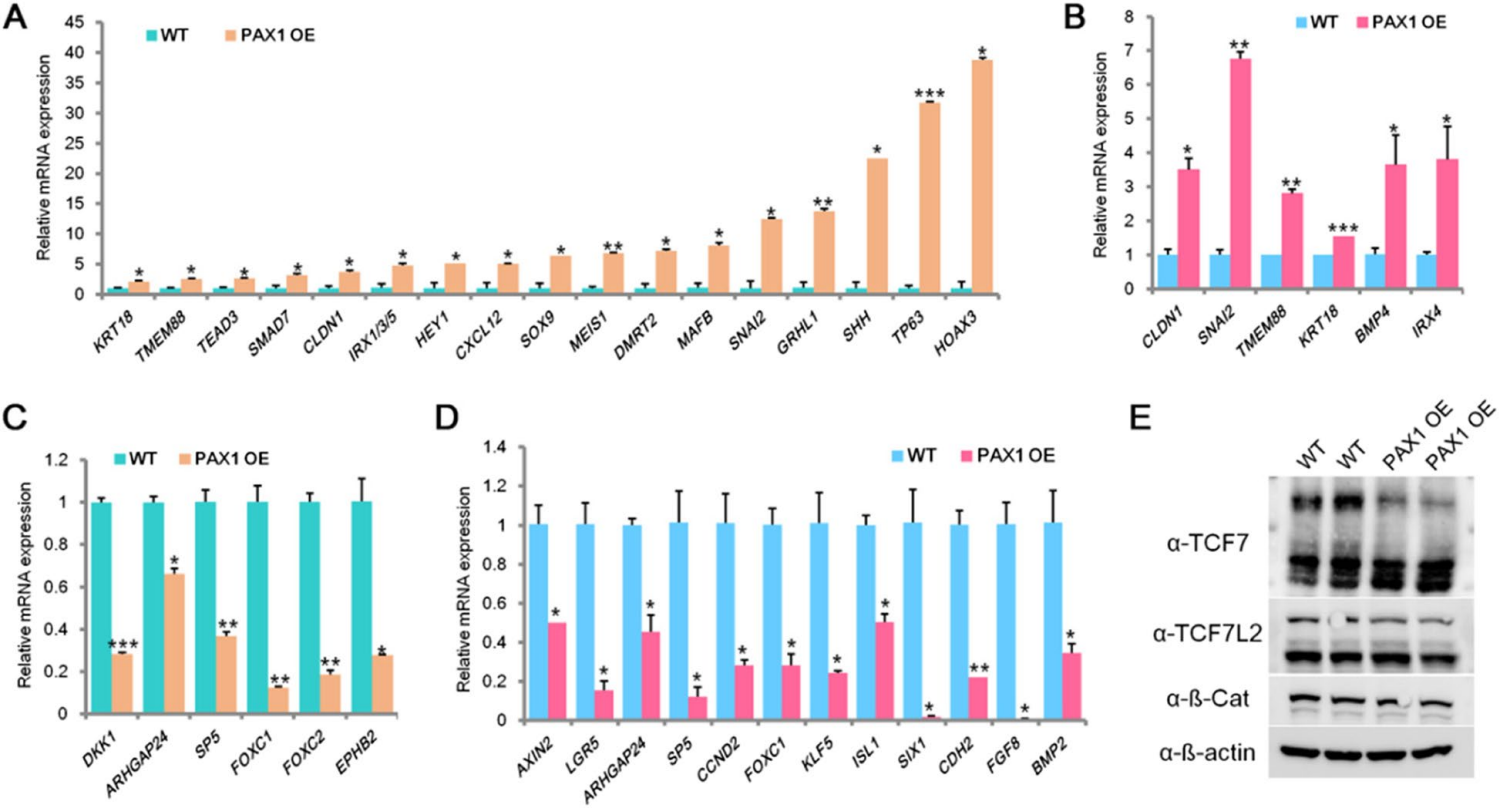
PAX1 represses Wnt signaling pathway in foregut and pharyngeal endoderm cells. **A-B** qRT-PCR analyses of AFE and PE associated genes in hESC-derived AFE (A) and PE (B) cells. AFE: anterior foregut endoderm; PE: pharyngeal endoderm. PAX1 OE: PAX1 overexpression. **C-D** qRT-PCR analyses performed on known Wnt target genes in hESC-derived AFE (C) and PE (D) cells. **E** Western blotting to analyze the protein levels of indicated proteins in WT and PAX1 OE hESC-derived PE cells. ß-actin was used as loading control

Interestingly, PAX1 has been reported to be essential for pharyngeal pouch morphogenesis and pharmacological activation of Wnt signaling pathway hinders the formation of pharyngeal pouches [18, 28, 70]. In line with this, we observed significant upregulation of foregut and pharyngeal endoderm associated genes [13, 18, 21, 71, 72] in PAX1 OE cells (Fig. 6A and B). Moreover, we also observed consistent downregulation of known canonical Wnt target genes in both foregut and pharyngeal endoderm cells (Fig. 6C and D), gives a hint that PAX1 promotes foregut and pharyngeal endoderm differentiation via suppressing Wnt signaling. To determine whether PAX1 represses Wnt signaling via regulation on TCFs during endoderm differentiation, we tested the levels of TCFs in human ESC derived pharyngeal endoderm cells (Fig. 6E). As TCF7 and TCF7L2 are highly enriched on the critical target genes as analyzed by ChIP-seq datasets during endoderm differentiation (Fig. 5D), we examined the protein levels of TCF7 and TCF7L2 which showed significant decrease when PAX1 was ectopically expressed (Fig. 6E), consistent with our results in HEK293FT and HCT116 cells. Altogether, these results support our finding that PAX1 inhibits Wnt signaling pathway through its regulation on TCFs in pharyngeal endoderm differentiation.

### PAX1 mutations found in SCID patients significantly compromise its ability in repressing canonical Wnt signaling pathway

**Fig. 7:**
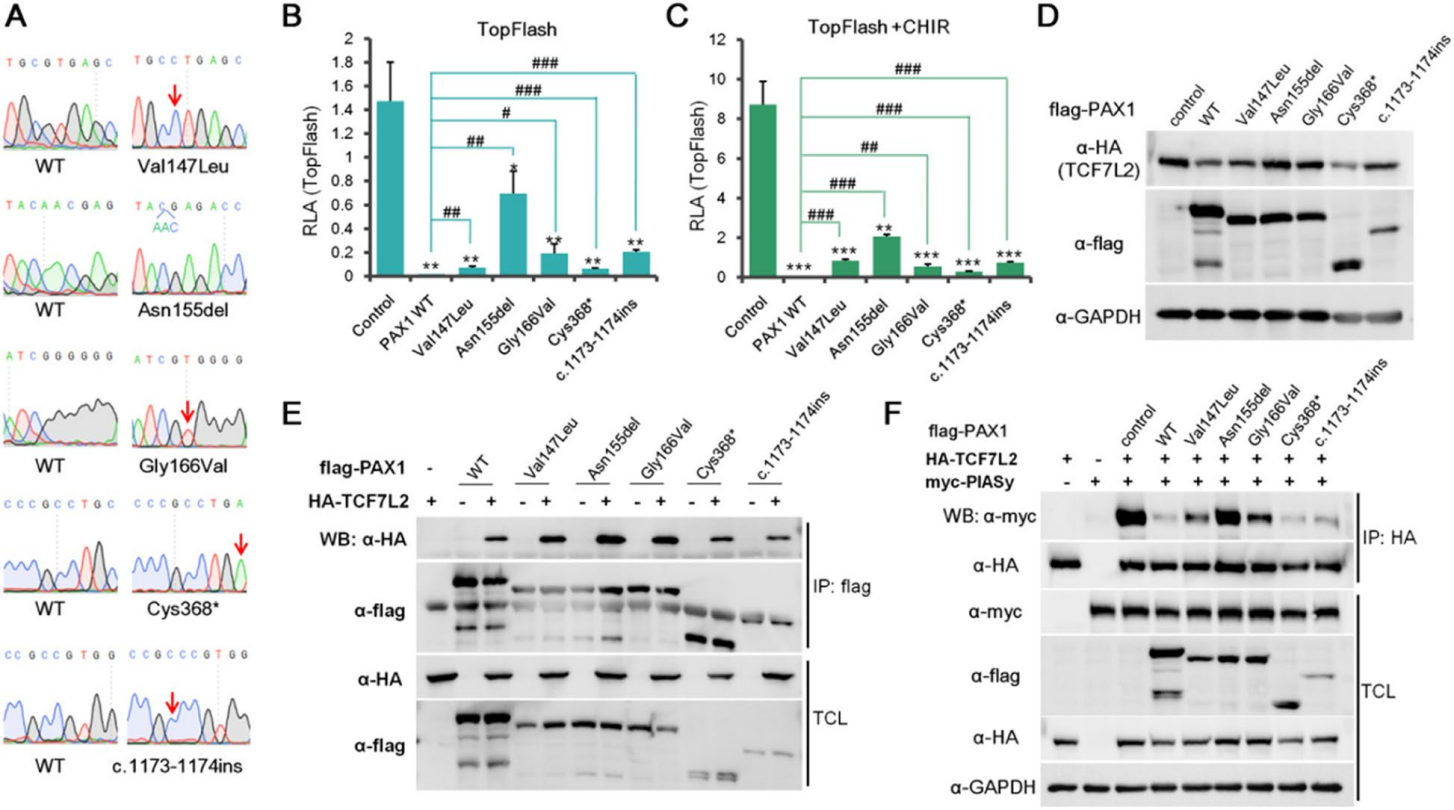
The roles of PAX1 mutants associated with SCID in regulation of Wnt signaling pathway. **A** Sanger sequencing results of wild type (WT) control and SCID associated PAX1 mutation constructs. **B-C** TopFlash luciferase reporter assays performed in HEK293FT cells transfected with empty vector control, WT or PAX1 mutation constructs without (B) or with (C) treatment of CHIR99021 (CHIR). * represents significant decrease compared with control, while # represents significant increase compared with PAX1 WT. **D** HEK293FT cells were transfected with HA-tagged TCF7L2 and empty vector control, PAX1 WT or PAX1 mutation construct (flag-tagged). TCF7L2 levels were analyzed by western blotting with anti-HA antibody. GAPDH was used as loading control. **E** A series of PAX1 mutation constructs with flag-tag were co-transfected with HA-tagged TCF7L2 in HEK293FT cells. Co-IP and western blotting assays were performed to test the interactions between TCF7L2 and PAX1 proteins. **F** Interactions between TCF7L2 and PIASy were assessed by co-IP and western blotting with empty vector control, PAX1 WT or PAX1 mutation constructs cotransfection in HEK293FT cells. Co-IP was done with anti-HA antibody. TCL and IP samples were assayed by western blotting using anti-myc antibody for PIASy and anti-flag antibody for PAX1 proteins, respectively. GAPDH was used as loading control

During embryogenesis, the thymus arises from the endoderm of the third pharyngeal pouch and PAX1 is known to be essential for thymus development [21, 47]. Definitive endoderm specification is the essential early and one of the most important steps to the generation of thymus epithelial cells [25]. Notably, there are quite some PAX1 mutants identified in Severe Combined Immunodeficiency (SCID) patients with Otofaciocervical Syndrome Type 2 (OTFCS2) [20, 21]. To test whether these mutants affect the role of PAX1 in suppressing canonical Wnt signaling pathway, we cloned Val147Leu, Asn155del, Gly166Val, Cys368*, c.1173-1174ins mutants [20, 21] into flag tag-fused expression plasmid and confirmed the success of cloning by Sanger sequencing (Fig. 7A). Then we tested the roles of these PAX1 mutants in Wnt signaling pathway with TopFlash reporter assay in HEK293FT cells. The results showed that PAX1 mutants can still repress TopFlash activity with or without CHIR99021 treatment, but the suppressing levels are significantly compromised to different extents (Fig. 7B-C). Given that the decreasing levels of TCF7L2 protein are consistent with that of TopFlash activity caused by different PAX1 mutants ectopically expressed (Fig. 7B-D), we could not exclude that the decreasing of TopFlash activity could be partly due to the reduction of TCF7L2 protein level, even though we can still observe significant repression of TopFlash activity in Asn155del mutant overexpressed group, in which almost no changes of TCF7L2 protein level were shown (Fig. 7B-D and Fig. S5A-D). Interestingly, it can still bind to TCF7L2 comparable to WT PAX1 (Fig. 7E). Importantly, we further checked their ability of competing with PIASy in interacting with TCF7L2 and observed consistent compromising inhibition levels of PIASy and TCF7L2 association (Fig. 7F). Taken together, our data indicated that PAX1 mutants found in SCID patients (Val147Leu, Asn155del, Gly166Val, Cys368*, c.1173-1174ins) significantly compromise its role in repressing canonical Wnt signaling pathway by perturbing its ability of modulating TCF7L2 SUMOylation.

## Discussion

In this study, we showed that PAX1 plays a repressing role in canonical Wnt signaling pathway by interacting with TCFs. We further demonstrated that PAX1 affects the transcriptional activity and protein stability of TCF7L2 via reducing its SUMOylation level. Moreover, we found that the suppressing role of PAX1 on Wnt signaling pathway results in impaired definitive endoderm differentiation and enhanced foregut and pharyngeal endoderm differentiation, in line with the reported functions of canonical Wnt signaling during endoderm differentiation. In summary, our study has revealed a novel role of PAX1 in regulation of canonical Wnt signaling pathway and a new molecular mode of action of PAX1 in endoderm differentiation.

As a member of the same subgroup of PAX1 gene family, PAX9 has been shown to play synergistic roles with PAX1 in vertebral column development and chondrogenic differentiation of sclerotomal cells [20]. Previous study reported that PAX9 can modulate Wnt signaling in murine palatogenesis by binding to regions near the transcription start sites of Dkk1 and Dkk2 as well as the intergenic region of Wnt9b and Wnt3 ligands [45]. Another group showed a genetic interaction of Pax9 with Tbx1 and Gbx2 in the pharyngeal endoderm to contribute to cardiovascular development in mouse embryos [73, 74]. However, no studies reported the role of PAX1 in Wnt signaling pathway so far and its molecular mode of action in pharyngeal pouch endoderm is far from well-defined. Here, we revealed a new function of PAX1 in repressing canonical Wnt signaling pathway with different molecular roles from reported functions of PAX9, by interacting with TCF7L2 and attenuating its post-translational modification, thus regulating the expression of Wnt downstream targets.

With a hESC-derived endoderm differentiation strategy and genome-wide transcriptome sequencing, we uncover that PAX1 regulates a group of known Wnt target genes in endoderm cells. We further show that PAX1 represses the expression of critical transcription factors and signaling molecules bound by TCFs that play essential functions in definitive endoderm formation, including *SOX17, FOXA2, EOMES, GATA4, GATA6, BMP2, FGF1, FGF10*, etc. [75–80]. Consistent with this, we observed impaired definitive endoderm differentiation. Whereas, in the later stage, PAX1 OE promotes foregut and pharyngeal endoderm formation by repressing the expression of Wnt target genes and activating foregut and pharyngeal endoderm associated genes, agrees with the roles of Wnt signaling in this process [13]. Importantly, there are more and more reports recent years showed PAX1 deficiency is associated with otofaciocervical syndrome type 2 (OTFCS2) patients displayed severe combined immunodeficiency (SCID) phenotypes due to altered thymus development [21–24, 81]. Thymus is a critical organ derived from pharyngeal endoderm in which PAX1 shows expression conserved in vertebrates [28, 47, 82, 83]. By construction of PAX1 SCID associated mutants, we show that the ability of PAX1 on repressing Wnt signaling is significantly compromised by different mutations compared with WT PAX1. Thus, our findings suggest that the function of PAX1 in regulation of canonical Wnt signaling pathway might play a role in thymus development, at least in part. The compromised function of PAX1 variants in regulation of Wnt signaling might be due to their altered protein conformation [21]. It would be interesting to know how the changes of even one amino acid alter the conformation and/or function of a protein in further studies.

## Conclusions

In conclusion, our study highlights a new function of PAX1 in the regulation of canonical Wnt signaling pathway and endoderm differentiation, and provides exciting novel insights into the molecular mode of action of PAX1 and the molecular machinery orchestrating embryogenesis and congenital diseases as well.

### Supplementary Information

**Supplementary Material 1.**

**Supplementary Material 2.**

## Abbreviations

PAX1: Paired Box-1
PAX1 OE: PAX1 overexpression
SCID: Severe combined immunodeficiency
OTFCS2: Otofaciocervical syndrome type 2
hESC: Human Embryonic Stem Cell
DE: Definitive endoderm
AFE: Anterior foregut endoderm
PE: Pharyngeal endoderm
EMT: Epithelial-to-mesenchymal transition
MO: Morpholino
CHIR: CHIR99021
LiCl: Lithium Chloride
co-IP: Co-immunoprecipitation
EV: Empty vector
CHX: Cycloheximide
scRNA-Seq: single-cell RNA sequencing
ChIP-seq: Chromatin immunoprecipitation followed by sequencing
FACS: Fluorescence-activated cell sorting
qRT-PCR: Quantitative real-time PCR
WB: Western blotting
ß-Cat: ß-catenin
WT: Wild type
RLA: Relative luciferase activity
FL: Full length
TCL: Total cell lysate
UMAP: Uniform manifold approximation and projection
