# Supplementary material for: PAX1 represses canonical Wnt signaling pathway and plays dual roles during endoderm differentiation
Source: Cell Commun Signal. 2024 Apr 26;22:242. doi: 10.1186/s12964-024-01629-3 (PMC11046865; doi:10.1186/s12964-024-01629-3)
Supplement: Supplementary file 1 — Supplementary Material 1. [file 12964_2024_1629_MOESM1_ESM.docx]

**Supplementary Figures**

**
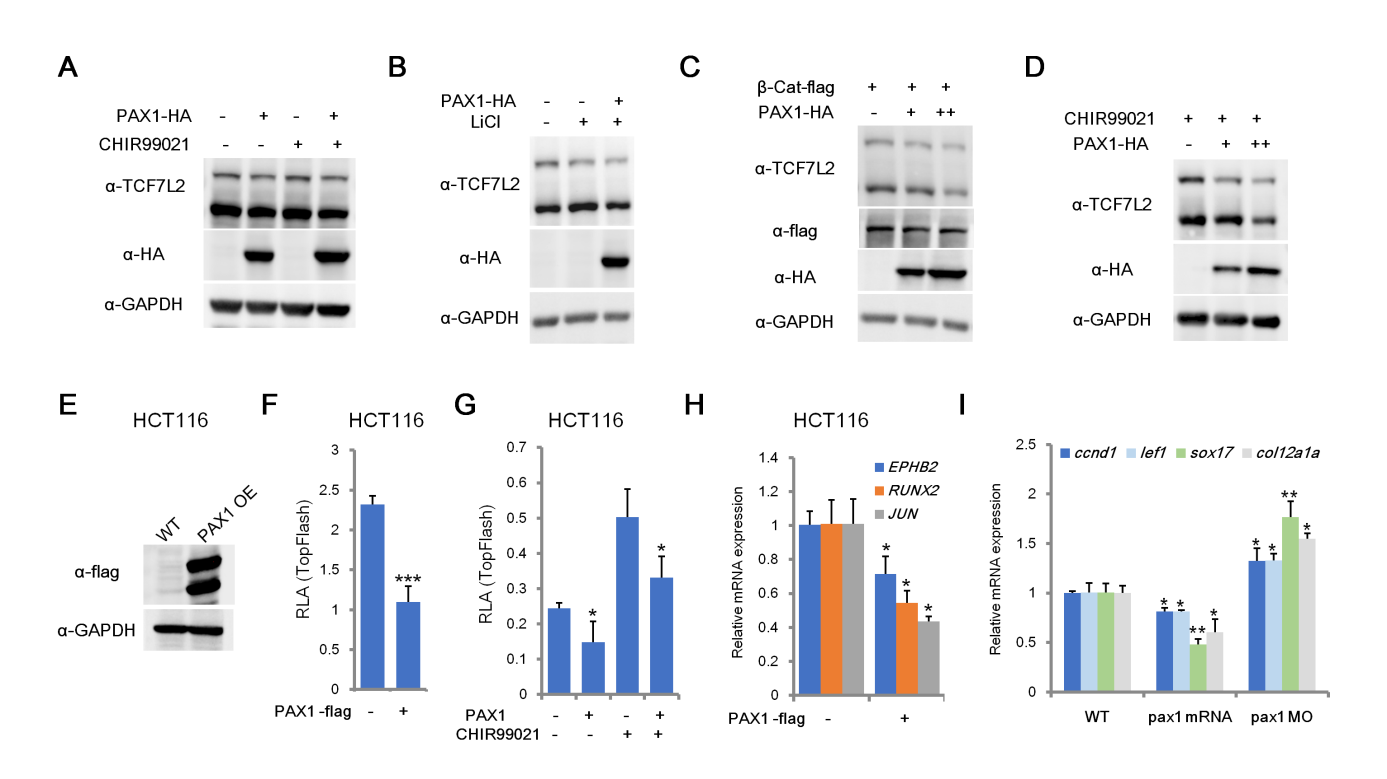
**

**Fig. S1 PAX1 represses canonical Wnt signaling in vertebrate cells.**

**(A-D)** Western blotting tested the expression of TCF7L2 and transfected plasmids corresponding to samples in Fig. 1A-1D. Biological triplicates were done in each assay and representative images were shown. GAPDH was used as loading control.

(**E**) Western blotting to confirm the success of generation of flag-tag fused PAX1 overexpressed (OE) HCT116 cell lines.

**(F-G)** TopFlash luciferase reporter assays in control and PAX1 OE HCT116 cell lines with or without treatment of CHIR99021 (CHIR).

**(H)** qRT-PCR analyses of Wnt target genes performed in HCT116 cells.

**(I)** The mRNA expression levels of indicated wnt target genes in wild-type (WT) control, *pax1* mRNA or MO injected zebrafish embryos at 70% epiboly analyzed by qRT-PCR. Values are mean ± SD (n=2; around 40 embryos randomly harvested for each group). **P*<0.05; ***P*<0.01; ****P*<0.001.

**
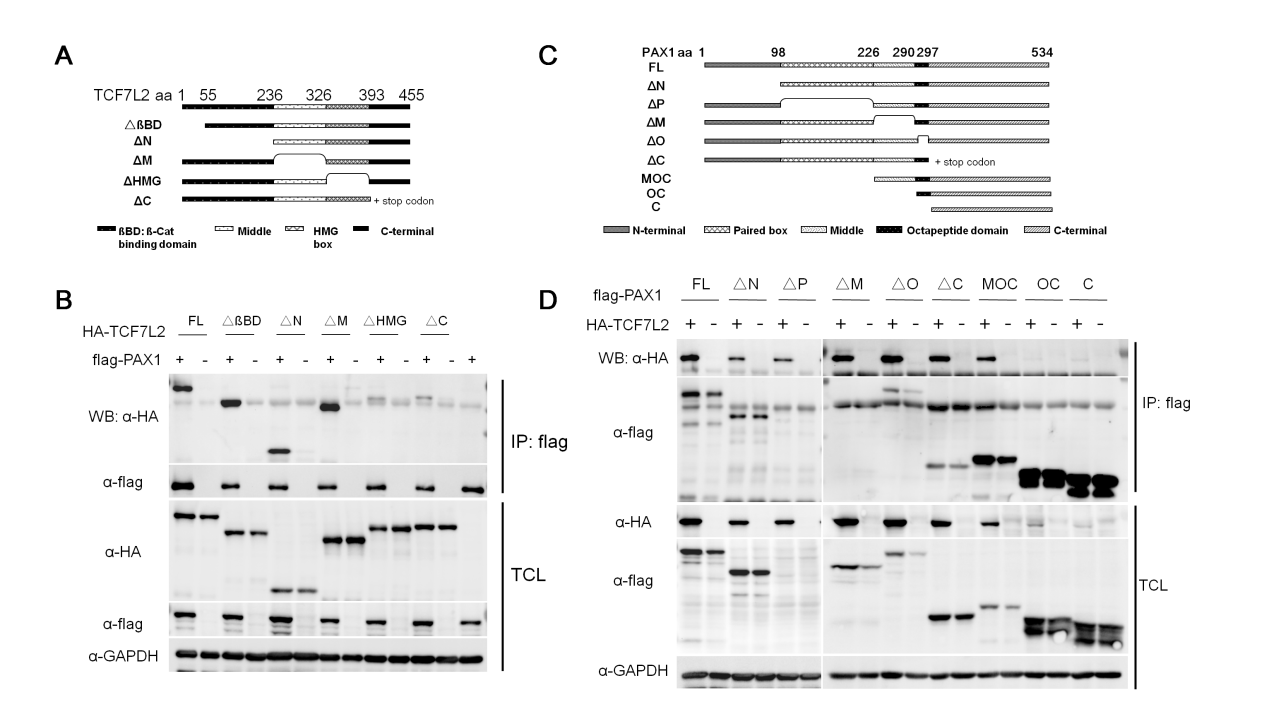
**

**Fig. S2** [**Domain mapping required for the interaction between**](https://www.researchgate.net/figure/Domain-mapping-required-for-the-interaction-between-EPRS-and-PCBP2-a-Full-length-EPRS_fig4_307871221) **PAX1 and TCF7L2.**

**(A-B)** A series of TCF7L2 deletions with HA-tag depicted with scheme in (A) were co-transfected with flag-tagged PAX1 in HEK293FT cells. Protein extracts were immunoprecipitated with M2 beads (with flag antibody). Total cell lysates (TCL) and immunoprecipitates (IP) were analyzed by western blotting with both anti-HA (α-HA) antibody for TCF7L2 proteins and flag (α-flag) antibody for PAX1 (B).

**(C-D)** Schematic diagram depicting different PAX1 deletions with flag-tag is shown in (C). Flag-tagged PAX1 deletions were co-transfected with HA-tagged TCF7L2 in HEK293FT cells and the interactions were assessed with co-IP and western blotting (D). FL: full length.

**
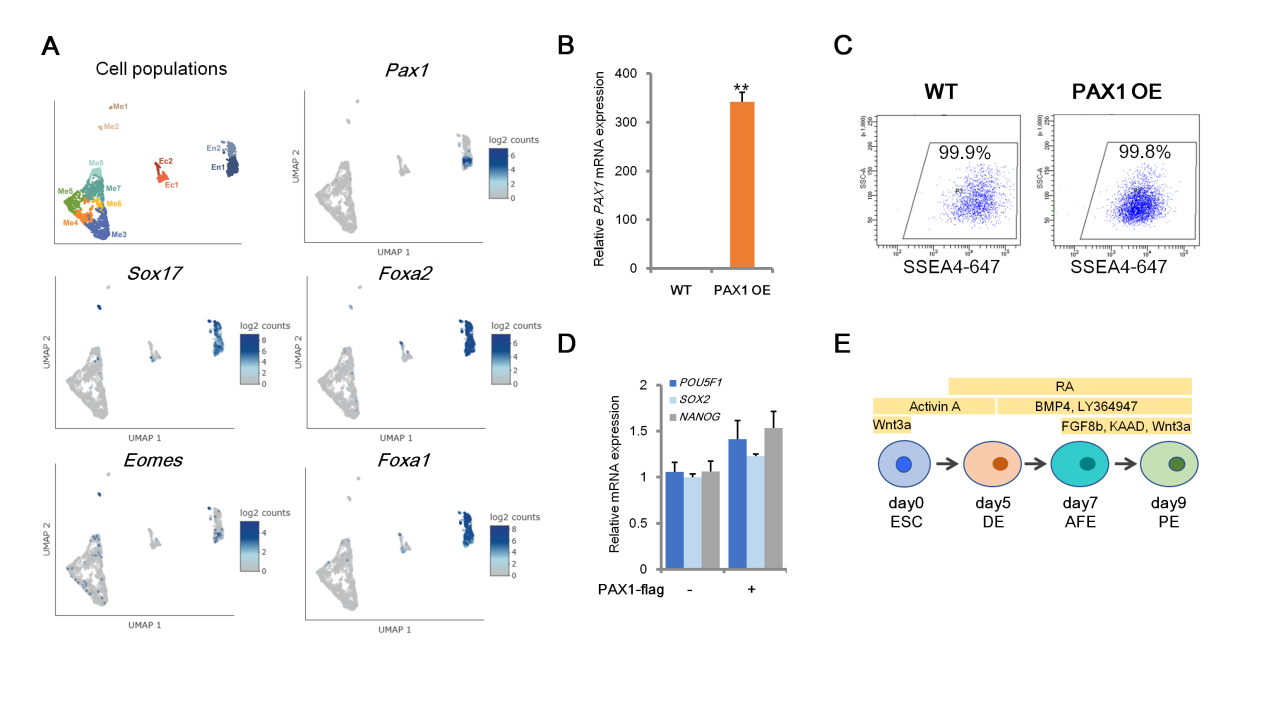
**

**Fig. S3 *Pax1* is co-expressed with definitive endoderm marker genes in mouse embryos and PAX1 overexpression in hESC does not affect its pluripotency.**

**(A)** UMAP plots showing the expression of Pax1 and endoderm marker genes in different cell populations (indicated by different colors), analyzed from the published scRNA-seq data (PRJEB14363) performed in isolated anterior cardiac region of E7.75 to E8.25 mouse embryos. **En1: definitive endoderm**; En2: yolk sac endoderm; Ec1: surface/amnion ectoderm; Ec2: neurectoderm; Me1-8: mesodermal cell types [including cardiac related (Me3-8); endothelial (Me2) and blood (Me1)].

**(B)** qRT-PCR analyses of the expression of PAX1 in WT and PAX1 overexpression (OE) hESC lines.

**(C)** Flow cytometry analysis of WT and PAX1 OE hESC lines stained for hESC surface marker SSEA4 (Alexa Fluor 647) shows PAX1 OE did not change the percentage of SSEA4^+^ population.

**(D)** qRT-PCR analyses of the expression of ESC pluripotency marker genes in control (-) and PAX1-flag OE (+) hESC lines.

**(E)** Scheme depicts endoderm differentiation protocol and stages used for analysis. ESC: embryonic stem cell. DE: definitive endoderm. AFE: anterior foregut endoderm. PE: pharyngeal endoderm.


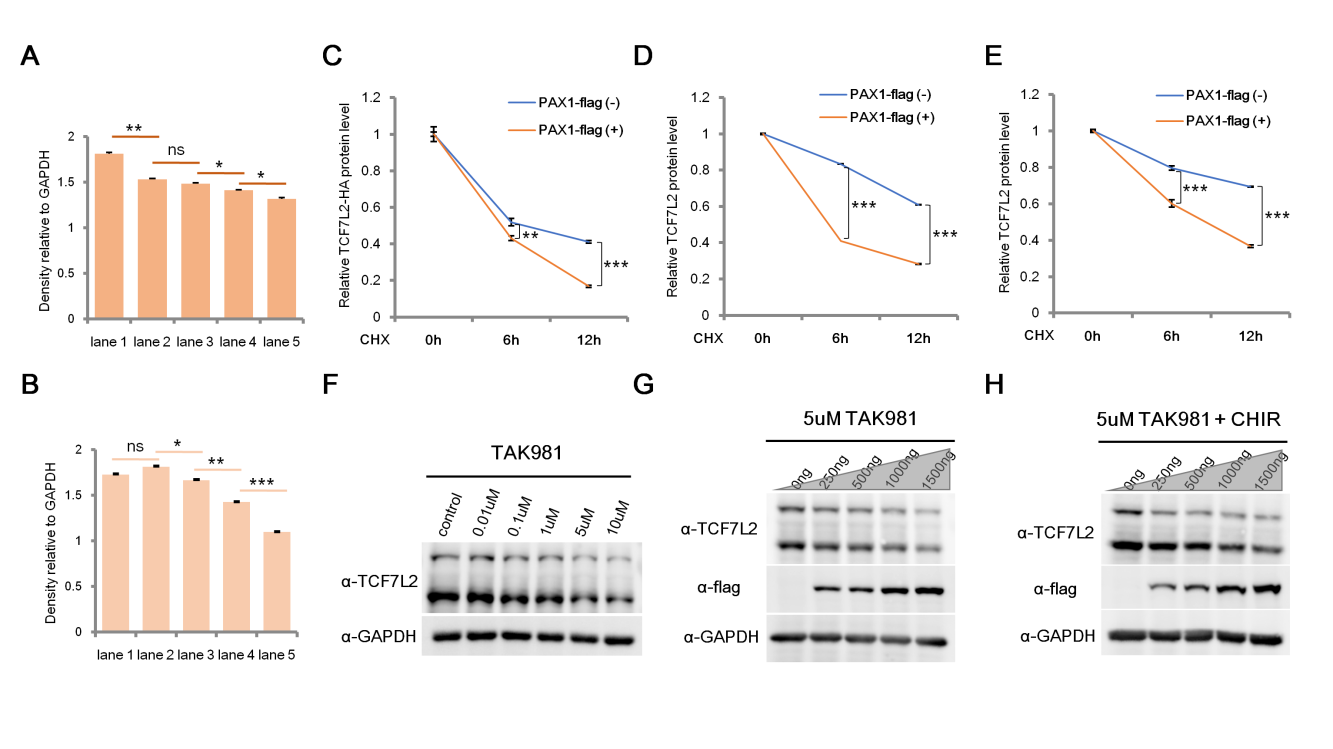


**Fig. S4 PAX1 reduces TCF7L2 protein level.**

**(A-B)** Densitometry analysis of western blots in Fig. 4A-4B (n = 2, mean ± SD. **P* < 0.05, ***P* < 0.01, ****P* < 0.001, ns: not significant).

**(C-E)** Quantification analysis of western blots in Fig. 4C-4E and density relative to GAPDH was shown. Density of CHX untreated (0h) samples were adjusted to 1 and relative HA/TCF7L2 protein levels at each time points were shown to compare HA/TCF7L2 protein half-life with (+) or without (-) PAX1 OE (n = 2, mean ± SD. **P* < 0.05, ***P* < 0.01, ****P* < 0.001).

**(F)** HEK293FT cells treated with a SUMO inhibitor (TAK981) with indicated concentrations for 48 hours and harvested for TCF7L2 level testing by western blotting. GAPDH was used as loading control.

**(G)** HEK293FT cells were transfected with increasing amount of PAX1-flag plasmid (0, 250, 500, 1000, 1500ng) and treated with 5uM TAK981 for 48 hours before harvested. Endogenous TCF7L2 levels were analyzed by western blotting. GAPDH was used as loading control.

**(H)** Same as (G) except cells were treated with 10μM CHIR99021 (+CHIR) for 24 hours before harvested.


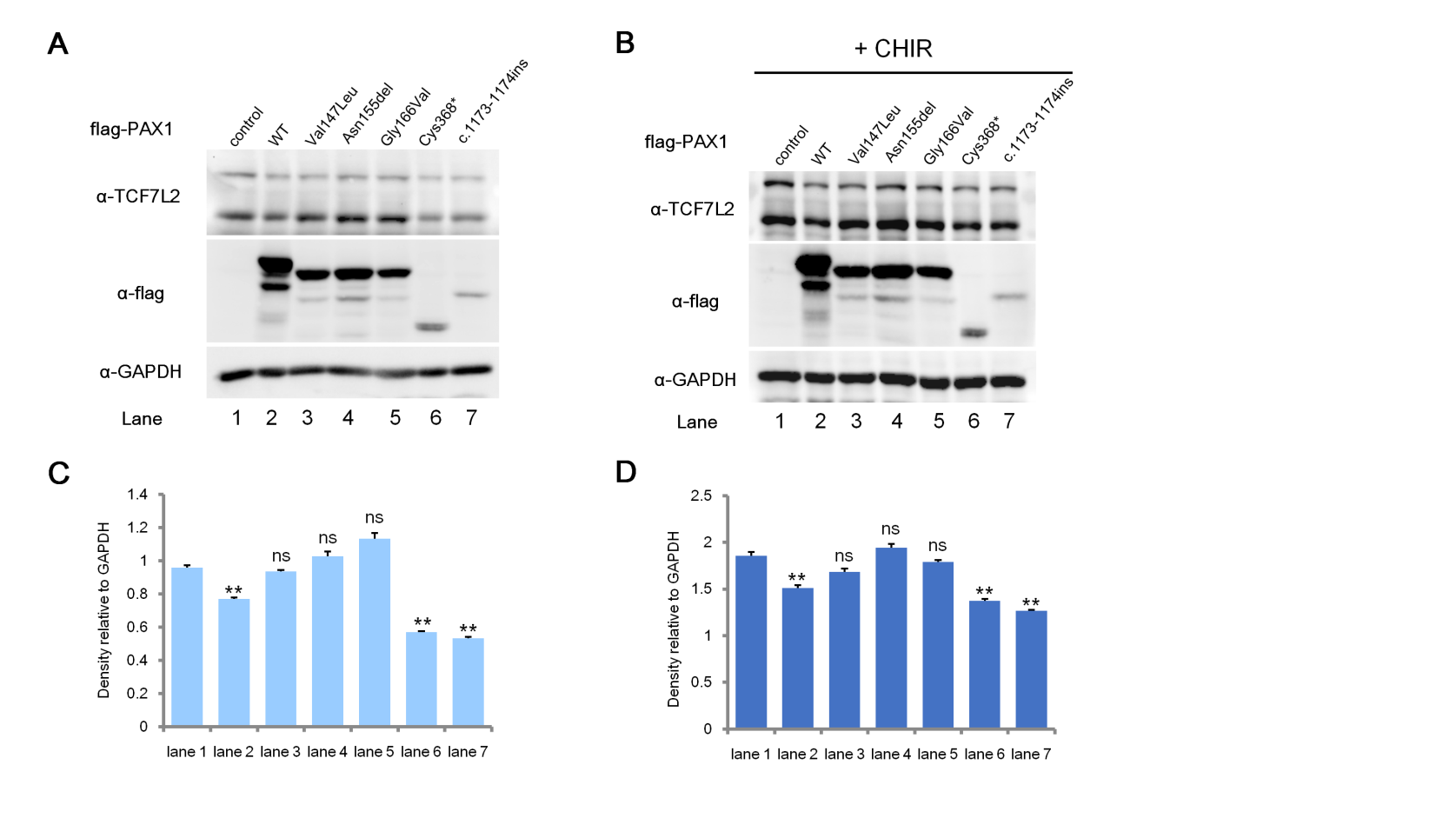


**Fig. S5 The roles of PAX1 mutants found in SCID patients in regulation of TCF7L2 protein level.**

**(A)** Western blotting tested the expression of TCF7L2 and transfected flag-tagged PAX1 plasmids corresponding to samples in Fig. 7B-7C. Biological triplicates were done in each assay and representative images were shown. GAPDH was used as loading control.

**(B)** Same as (A) except cells were treated with 10μM CHIR99021 (+CHIR) for 24 hours before harvested.

**(C-D)** Densitometry quantification analysis of western blots in panel A (C) and B (D). Statistical analysis was done for lane 2-7 compared with lane 1 (n = 2, mean ± SD. **P* < 0.05, ***P* < 0.01, ****P* < 0.001).

**Supplementary Tables**

**Table S1 List of primers used for qRT-PCR.**

| Genes | Forward primer | Reverse primer |
| --- | --- | --- |
| *GAPDH* | GTCTCCTCTGACTTCAACAGCG | ACCACCCTGTTGCTGTAGCCAA |
| *AXIN2* | AGTGTGAGGTCCACGGAAAC | CTTCACACTGCGATGCATTT |
| *LEF1* | AACATGGTGGAAAACGAAGC | GGGTTGGCAGTGATTGTCTT |
| *TBX3* | AAAACGCAAGGCACAAGGAC | GGCAAGGCGAAAAATCAGCA |
| *RUNX1* | CCTAGGGACGCCAGAAGGAA | GCACTTTCTCCCCCTGTTGT |
| *FOXM1* | CCAAAACCTGCAGCTAGGGA | CCATCACAGGTCTCCCGTTT |
| *EPHB2* | CGCCATCTATGTCTTCCAGGTG | GATGAGTGGCAACTTCTCCTGG |
| *RUNX2* | CCCAGTATGAGAGTAGGTGTCC | GGGTAAGACTGGTCATAGGACC |
| *JUN* | CCTTGAAAGCTCAGAACTCGGAG | TGCTGCGTTAGCATGAGTTGGC |
| *POU5F1* | GCCGCTGGCTTATAGAAGGT | ACATGGCATGCATACACACA |
| *SOX2* | GGATAAGTACACGCTGCCCG | ATGTGCGCGTAACTGTCCAT |
| *NANOG* | CAATGGTGTGACGCAGGGAT | TGCACCAGGTCTGAGTGTTC |
| *SOX17* | ACGCTTTCATGGTGTGGGCTAAG | GTCAGCGCCTTCCACGACTTG |
| *FOXA2* | GGAACACCACTACGCCTTCAAC | AGTGCATCACCTGTTCGTAGGC |
| *EOMES* | AAATGGGTGACCTGTGGCAAAGC | CTCCTGTCTCATCCAGTGGGAA |
| *GATA4* | GCGGTGCTTCCAGCAACTCCA | GACATCGCACTGACTGAGAACG |
| *GATA6* | GCCACTACCTGTGCAACGCCT | CAATCCAAGCCGCCGTGATGAA |
| *FOXA1* | GCTACTACGCAGACACGCAGGA | GGGTTGGCATAGGACATGTTGA |
| *LGR5* | ACCAGACTATGCCTTTGGAAAC | TCCCAGGGAGTGGATTCTATT |
| *KLF5* | GGAGAAACGACGCATCCACTAC | GAACCTCCAGTCGCAGCCTTC |
| *ARHGAP24* | ACCTCCGAGAACTTCCAGAACC | CCACTGGCAAACTCTTCACCTG |
| *CD44* | CCAGAAGGAACAGTGGTTTGGC | ACTGTCCTCTGGGCTTGGTGTT |
| *MMP2* | AGCGAGTGGATGCCGCCTTTAA | CATTCCAGGCATCTGCGATGAG |
| *CDH2* | CCTCCAGAGTTTACTGCCATGAC | GTAGGATCTCCGCCACTGATTC |
| *TWIST1* | GCCAGGTACATCGACTTCCTCT | TCCATCCTCCAGACCGAGAAGG |
| *FN1* | ACAACACCGAGGTGACTGAGAC | GGACACAACGATGCTTCCTGAG |
| *FOXC1* | AGCAGCAGAACTTCCACTCG | AGTCGTAGACGAAAGCTCCG |
| *FOXC2* | TCACCTTGAACGGCATCTACCAG | TGACGAAGCACTCGTTGAGCGA |
| *VIM* | AGGCAAAGCAGGAGTCCACTGA | ATCTGGCGTTCCAGGGACTCAT |
| *BMP2* | TGTATCGCAGGCACTCAGGTCA | CCACTCGTTTCTGGTAGTTCTTC |
| *FGF1* | ATGGCACAGTGGATGGGACAAG | TAAAAGCCCGTCGGTGTCCATG |
| *FGF10* | GTGCGGAGCTACAATCACCT | CTCCAGGATGCTGTACGGG |
| *SP5* | GAAACAGTGCTCGGGTTTTC | TAGCTCTGCATGGAGCTGAA |
| *ISL1* | TCCCTATGTGTTGGTTGCGG | CATTTGATCCCGTACAACCTGA |
| *PAX1* | TCGCTATGGAGCAGACG TATG | GCTGCCGACTGATGTCACA |
| *FGF8* | GGACACCTTTGGAAGCAGAGTC | CCAGCACAATCTCCGTGAAGAC |
| *SIX1* | GGAGGCCAAGGAAAGGGAGAAC | CCCCCTTCCAGAGGAGAGAG |
| *CCND2* | TACCTGGACCGTTTCTTGGC | TCCACTTCAACTTCCCCAGC |
| *DKK1* | CTGCAAAAATGGAATATGTGT | CTTCTTGTCCTTTGGTGTGA |
| *KRT18* | CATGCAAAGCCTGAACGACC | GTATTTGCGAAGATCTGAGCCC |
| *TMEM88* | ATCTTGCTACCCGCTGTCAC | GAGCGCAGGAACTGAGAGTG |
| *TEAD3* | TCGGACGAGAAAACAGGTGTC | ACCTTCTTCCGAGCTAGAACC |
| *SMAD7* | CAGGCATTCCTCGGAAGTCA | CATCTGGACAGTCAGTTGGT |
| *CLDN1* | GTCTTTGACTCCTTGCTGAATCTG | CACCTCATCGTCTTCCAAGCAC |
| *IRX1/3/5* | GATCGCTGTAGTGCCTTGGA | CAGATGGTTCTGGGGCCG |
| *HEY1* | TGTCTGAGCTGAGAAGGCTGGT | TTCAGGTGATCCACGGTCATCTG |
| *CXCL12* | CTCAACACTCCAAACTGTGCCC | CTCCAGGTACTCCTGAATCCAC |
| *SOX9* | AGGAAGCTCGCGGACCAGTAC | GGTGGTCCTTCTTGTGCTGCAC |
| *MEIS1* | GCATGGGCTCCTCTGTCAAT | GGGAAGAGGGGGTGTCCATA |
| *DMRT2* | TTCGAGCGCAAAGCTGTGTACC | TCCCTCCTACATAAGTCTCCGC |
| *MAFB* | AGACGCCTACAAGGTCAAGTGC | CGACTCACAGAAAGAACTCGGG |
| *SNAI2* | ATCTGCGGCAAGGCGTTTTCCA | GAGCCCTCAGATTTGACCTGTC |
| *GRHL1* | AACCGCAGCAACAAGCCTGTGC | TGAGAGGGAAGCAGTGGCACTT |
| *SHH* | CCGAGCGATTTAAGGAACTCACC | AGCGTTCAACTTGTCCTTACACC |
| *TP63* | AACGGTGATGGTACGAAGCG | CATAAGTCTCACGGCCCCTC |
| *HOXA3* | GCAGCTCCAGCTCAGGCGAA | GCCGGCACAGGTAGCGGTTG |
| *BMP4* | CGGAAGCTAGGTGAGTGTGG | TCGAGATAGCTTGGACGGGA |
| *IRX4* | CGCCTTCTACTCGCTGAACA | AGAGCTGGCTCGTAAGGGTA |
| *ccnd1* | AAGTGGCTGCCTGTCTTGTT | ACTGCTGATTGTAGCTGCGT |
| *lef1* | GGAGCCCAAAAGACCTCACA | TTCCTTGCGGGCTAATTCGT |
| *sox17* | CGCTTCCTGGGATGTGTGAT | AGCACTCATACCTTCCGTGC |
| *col12a1a* | GCTACCCAGGTTTTGCGTAGT | GTTTGACATGCGTCCTCAGC |
| *rpl13a* | AGGCTGAAGGTGTTTGATG | TTTCAGACGCACAATCTTGA |

**Table S2 Differentially expressed genes in RNA-seq data of day5 definitive endoderm.**
